# Animal amino acid sensor — A review

**DOI:** 10.5713/ab.24.0366

**Published:** 2024-08-26

**Authors:** Yongding Ke, Xinyu Peng, Chengchuang Song, Xingtang Fang, Yanhong Wang, Chunlei Zhang

**Affiliations:** 1 School of Life Science, Jiangsu Normal University, Jiangsu 221116, China

**Keywords:** Amino Acid Sensor, Livestock, Mammalian Target of Rapamycin

## Abstract

Cell growth and metabolism necessitate the involvement of amino acids, which are sensed and integrated by the mammalian target of rapamycin complex 1 (mTORC1). However, the molecular mechanisms underlying amino acid sensing remain poorly understood. Research indicates that amino acids are detected by specific sensors, with the signals being relayed to mTORC1 indirectly. This paper reviews the structures and biological functions of the amino acid sensors identified thus far. Additionally, it evaluates the potential role these sensors play in the developmental changes of the livestock production.

## INTRODUCTION

Amino acids are fundamental for protein and peptide synthesis and act as molecular signal transducers that regulate various metabolic processes in animals [1]. Leucine and arginine are among the amino acids most thoroughly studied, with the molecular mechanisms of their specific sensors, including Sestrins and CASTOR1/2, relatively well understood. Other proteins, such as MAP4K3 [2] and the G protein-coupled receptors T1R1/T1R3 [3], act as nonspecific amino acid binding sites and regulate the lysosomal localization of mTORC1 through mechanisms yet to be identified. The mammalian target of rapamycin (mTOR) is an exceptionally conserved serine/threonine kinase that orchestrates the regulation of nutrients, growth factors, and hormones essential for cell growth, proliferation, and survival. The mTOR is a conserved amino acid kinase composed of two distinct complexes: mTOR complex 1 (mTORC1) and mTOR complex 2 (mTORC2), which are involved in regulating cell growth, proliferation, and survival [4,5]. The mTOR pathway integrates multiple amino acid receptors and is important for nutrient perception in the body. The study of the molecular mechanism of amino acid sensing is helpful to improve the rate of amino acid absorption and transformation of the body, which plays a role in promoting the production of livestock. This paper discusses the molecular binding mechanisms of specific amino acid sensors and analyzes their significance in livestock health and production.

## AMINO ACID SPECIFIC SENSOR

### Arginine sensor

Arginine, a ubiquitous amino acid in animals, serves multiple vital functions beyond being a precursor for protein synthesis; it is also essential for the synthesis of nitric oxide, urea, and glutamic acid. The mTORC1 signaling pathway, activated by arginine, plays a crucial role in various aspects of cell metabolism. This includes insulin secretion, intestinal cell migration [6], and muscle protein synthesis [7]. Recent discoveries have identified specific arginine sensors on the mTORC1 signaling pathway, such as solute carrier family 38 member A9 (SLC38A9), cellular arginine and serine-threonine kinase 1 (CASTOR1), and transmembrane 4 L six family member 5 (TM4SF5). These sensors are instrumental in modulating the pathway’s response to arginine, highlighting its broad impact on cellular functions.

#### SLC38A9

As previously discussed, lysosomes serve as critical sites for binding and sensing amino acids and growth factors, thereby activating mTORC1. This leads to a speculative model wherein the lysosomal cavity contains an amino acid pool that is monitored by mTORC1. It is hypothesized that there are proteins located on the lysosomal membrane that facilitate the transfer of amino acids to other components on the lysosomal surface. SLC38A9 is one such protein that plays a key role in this proposed mechanism.

SLC38A9, a member of the sodium-coupled amino acid transporter family, solute transporter 38, is characterized by 11 transmembrane domains that detect amino acid signals. Distinctively, it also features an N-terminal cytoplasmic tail composed of 119 amino acids [8], setting it apart from other members of the SLC38 family. Mutations within its conserved domains impair its ability to bind to the Rag-Ragulator complex [9]. Through rapid lysosome purification and metabolite analysis, it has been observed that the concentration of arginine within the lysosome is significantly lower than the dissociation constant required for SLC38A9 transport. This discrepancy suggests that arginine regulation is governed by multiple mechanisms. Analytical data indicate that arginine at semi-maximal concentrations of 100–200 μM promotes the binding of full-length SLC38A9 to the Rag-Ragulator via its N-terminal cytoplasmic domain, facilitating the translocation of mTORC1 to the lysosomal surface, where it is activated by Rebsamen and Superti-Furga [10]. The knockout of SLC38A9 results in defective mTORC1 activation by amino acids, particularly arginine; in such cases, mTOR can still localize to the lysosomal surface but remains inactive in the presence of arginine and does not return to the cytoplasm in the absence of arginine [11]. This evidence confirms that SLC38A9 functions as the presumed arginine sensor on the lysosomal surface.

While the role of SLC38A9 as an arginine sensor may not seem critical due to its low affinity for arginine and uncertain arginine-binding capabilities, its function extends beyond simple ligand interaction. SLC38A9 acts similarly to Ragulator, serving as a guanine nucleotide exchange factor (GEF) for Rag GTPase. The binding of lysosomal arginine to SLC38A9 triggers an allosteric change, enabling the N-terminal domain of SLC38A9 to bind to the inactive Rag GTPase, independent of Ragulator. This interaction stimulates SLC38A9’s GEF activity, facilitating the displacement of guanosine diphosphate from RagA, which in turn allows guanosine triphosphate (GTP) to bind, activating RagA. Once RagA is activated, its affinity for SLC38A9 decreases, leading to the disassociation of SLC38A9 and allowing it to initiate another cycle of catalysis on the inactive Rag GTPase heterodimer [12]. This cyclical process suggests that SLC38A9, in the presence of arginine, activates multiple Rag GTPases and segregates Rag GTPase activation from mTORC1 recruitment. The detachment of the activated Rag GTPase from SLC38A9 is thus a critical step in the activation of mTORC1.

In addition to serving as an arginine sensor, SLC38A9 also facilitates the transport of other amino acids, notably leucine, in an arginine-mediated manner. Interestingly, lysine can promote the binding of SLC38A9 to Rag-Ragulator; Xhowever, this effect is considerably less pronounced than that of arginine, attributed to the low physiological correlation between lysine and SLC38A9. To validate these hypotheses and gain deeper insights into the mechanisms underlying these interactions, further analysis of SLC38A9’s crystal structure is essential.

SNAT2, encoded by the *SLC38A2* gene, is a member of the amino acid transporter family within the amino acid system. Lee et al [13] have experimentally demonstrated that inhibition of SNAT2 in L6 muscle cells leads to depletion of cellular glutamine, which in turn results in the depletion of other amino acids, notably leucine, thereby reducing mTORC1 activation. Furthermore, scientists have suggested that SNAT2 may act as an amino acid receptor, capable of sensing and signaling amino acid availability to mTORC1 [14–16]. However, the specific molecular mechanisms through which SNAT2 monitors extracellular amino acid availability and influences mTORC1 activation remain to be elucidated.

#### CASTOR1

CASTOR1/2 (cellular arginine sensor for mTORC1), encoded by the *GATS* protein gene, previously known as GATSL3/2 [17], plays a crucial positive regulatory role in the mTORC1 signaling pathway. Sequence analysis has revealed that CASTOR1 and CASTOR2 share 63% sequence homology in human cells and both contain two aspartic converting kinases, complex mutant enzymes, and TyrA (ACT) domains. The ACT domain is pivotal in binding small molecules such as amino acids and nucleotides. Sodium dodecyl sulfate-polyacrylamide gel electrophoresis experiments have demonstrated that CASTOR1 and CASTOR2 can form homologous and heterologous dimers in a 1:1 ratio, but only the Castor1-Castor1 and Castor1-Castor2 dimers can bind to arginine. In the absence of arginine, the CASTOR1 dimer binds to GATOR2, whereas the re-addition of arginine results in the release of GATOR2 from CASTOR1 [18]. Subsequent experiments indicated that arginine disrupts the GATOR2-CASTOR1 interaction by directly binding to CASTOR1 [19], thereby activating the mTORC1 pathway. Knockouts of CASTOR1 render mTORC1 insensitive to arginine, and CASTOR1 mutants unable to bind arginine fail to transmit arginine signals to mTORC1.

ACT1 and ACT3 display typical topologies, whereas ACT2 and ACT4 exhibit discontinuous regions with unusual topologies, featuring arginine binding pockets located at their interaction interfaces. For the regulatory process: firstly, CASTOR1 must form a dimer to effectively inhibit GATOR2 in the absence of arginine. Secondly, arginine is secured within the binding pocket through hydrogen bonds and hydrophobic forces with key residues [9], ensuring the specific binding and recognition between CASTOR1 and arginine. Finally, the arginine-CASTOR1 complex facilitates allosteric modulation of the adjacent GATOR2 binding sites. This modulation triggers the dissociation from GATOR2, thereby activating the mTORC1 signaling pathway.

#### TM4SF5

TM4SF5, an N-glycosylated tetramer, is comprised of four transmembrane (TM) domains, two extracellular loops, one intracellular loop, and two cytoplasmic terminal tails, structurally similar to transmembrane tetraproteins and is highly expressed in liver cancer cells. In the presence of arginine, TM4SF5 transitions from the plasma membrane to the lysosomal membrane where it interacts with mTOR and SLC38A9, facilitating the activation of the mTOR/S6K1 pathway. Notably, TM4SF5 can also bind arginine via its large extracellular loop domain, allowing arginine to be transported to the cytoplasm through SLC38A9, further activating mTOR/S6K1. Intriguingly, TM4SF5 and CASTOR1 share the same binding site on the TM4SF protein; in the absence of arginine, TM4SF5 binds to CASTOR1, thereby inhibiting the mTOR pathway. Conversely, the reintroduction of arginine permits TM4SF5 to bind to mTOR, activating the mTOR/S6K1 pathway [20]. However, additional research is necessary to fully elucidate the interaction mechanisms between TM4SF5 and the components of mTORC1, and to confirm TM4SF5’s role as an arginine sensor.

### Leucine sensor

Leucine serves not only as an amino acid for protein synthesis but also as a signaling molecule that directly influences various aspects of animal physiology [21]. A key mediator of leucine’s role as a signaling molecule is mTORC1, which is regulated by leucine sensors such as Sestrin2 and LRS.

#### Sestrin

**Figure 1:**
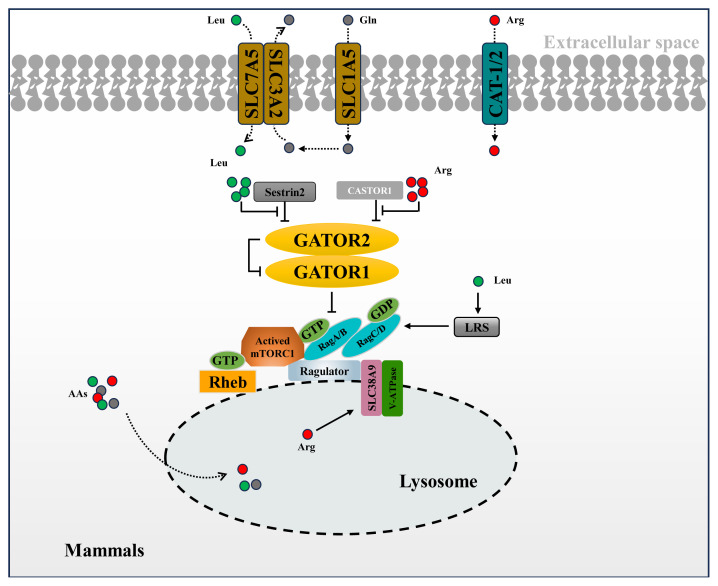
Amino acid transport diagram in mammalian cells. The figure depicts the transport of leucine, arginine, and glutamate from extracellular to intracellular and the activation of the associated signaling pathways. Among them, leucine enters cells through SLC7A5 and activates mTOR pathway via Sestrin2-GATOR2 pathway. Arginine enters the cell via CAT-1/2 and activates the mTOR pathway via the CASTOR1-GATOR2 pathway. mTOR, mammalian target of rapamycin.

Sestrin has three homologous complexes, namely Sestrin1/2/3, which are soluble proteins [22]. Sestrin2, characterized by its all-α-helical globular structure, features distinct N-terminal and C-terminal domains connected by a partially disordered, helical linker [23]. Equilibrium binding experiments and thermal transfer analysis have shown that leucine promotes the dissociation of Sestrin2 from GATOR2 by binding directly to Sestrin2 and inducing a conformational change. This disruption further facilitates mTORC1 activation by regulating upstream factors such as GATOR1 and Rag GTPase [24,25]. Notably, mutant Sestrin2, which cannot bind leucine, renders mTORC1 insensitive to leucine concentration changes and diminishes the lysosomal localization of mTORC1, without affecting RagGTPase [26]. These findings confirm Sestrin2’s role as a cytoplasmic leucine sensor. Recent structural analyses have elucidated the leucine-binding mechanism of Sestrin2, revealing a single leucine molecule binding site within the C-terminal domain. Additionally, studies have identified SH3 domain binding protein 4 (SH3BP4) as a negative regulator that mediates signal transduction between the RagGTPase complex and mTORC1 [27,28], offering another potential pathway for Sestrin2-mediated leucine signaling (Figure 1).

Earlier studies have shown that while Sestrin2 and GATOR2 bind effectively, this interaction is strengthened during amino acid starvation, and the addition of leucine disrupts this binding [29]. However, during leucine starvation, Sestrin2 can be phosphorylated by ULK1, potentially influencing mTORC1 activation in a classic positive feedback loop [30]. Moreover, under normal culture conditions with adequate leucine and other amino acids, Sestrin2 has been shown to activate AMPK and inhibit mTORC1 [31]. There may be other mechanisms of leucine perception that need to be further revealed. At the same time, whether there are differences in the ability of Sestrin2 to sense leucine in different tissues remains to be investigated [32,33].

#### Leucyl-tRNA synthetase

Leucyl-tRNA synthetase (LRS) belongs to the class of aminoacyl-tRNA synthetases (ARSs) characterized by a Roman fold, a large inserted CP1 domain, and a tRNA-binding anticodon region [34,35]. Additionally, LRS is part of the multi-tRNA synthetase complex (MSC), where its C-terminal extension plays a crucial role in binding other MSC components. In mammals, leucine triggers the translocation of LRS to the lysosomal surface, where it interacts with RagD and promotes GTP hydrolysis, suggesting that LRS functions as a GTPase-activating protein (GAP) for RagD [36]. The Michaelis constant for LRS-mediated leucine activation is 45 μM/L [37], and the interaction between leucine and LRS can desensitize mTORC1 to other amino acids. The absence of LRS renders mTORC1 insensitive to leucine, preventing its recruitment to the lysosomal surface. Moreover, LRS mutants that cannot bind leucine also result in diminished leucine sensitivity of mTORC1. These findings support the hypothesis that LRS is a potential intracellular leucine sensor within the amino acid-activated mTORC1 pathway. Interestingly, recent research has demonstrated that LRS can directly transmit intracellular leucine signals to mTORC1 through lysine 142 (K142) on RagA, with the leukocytosis of K142 promoting the formation of a RagA-GTP complex with high GTPase activity [38].

#### Acetyl-Coenzyme A

Recent studies have highlighted the role of leucine in regulating the mTOR pathway via its metabolite, acetyl-CoA, suggesting that acetyl-CoA might act as a leucine sensor. Specifically, leucine-derived acetyl-coenzyme A (AcCoA) positively influences mTORC1 by acetylating its regulator, Raptor, at K1097 [39]. This acetylation of Raptor is mediated by EP300 [40]. The relationship between leucine metabolism and mTORC1 activity is further mediated by intermediary enzymes, with AcCoA levels directly reflecting leucine abundance [41]. Furthermore, enoyl-CoA hydratase-1 (ECHS1), an enzyme involved in fatty acid oxidation, also plays a role in sensing activation of mTOR-related pathways [41]. Nutrient-induced acetylation of ECHS1 reduces its activity, leading to an accumulation of branched-chain amino acids (BCAAs), thereby activating mTOR signaling [22]. Thus, leucine-derived AcCoA regulates mTORC1 activity both through direct effects on Raptor and by influencing BCAA levels.

### Methionine sensor

**Figure 2:**
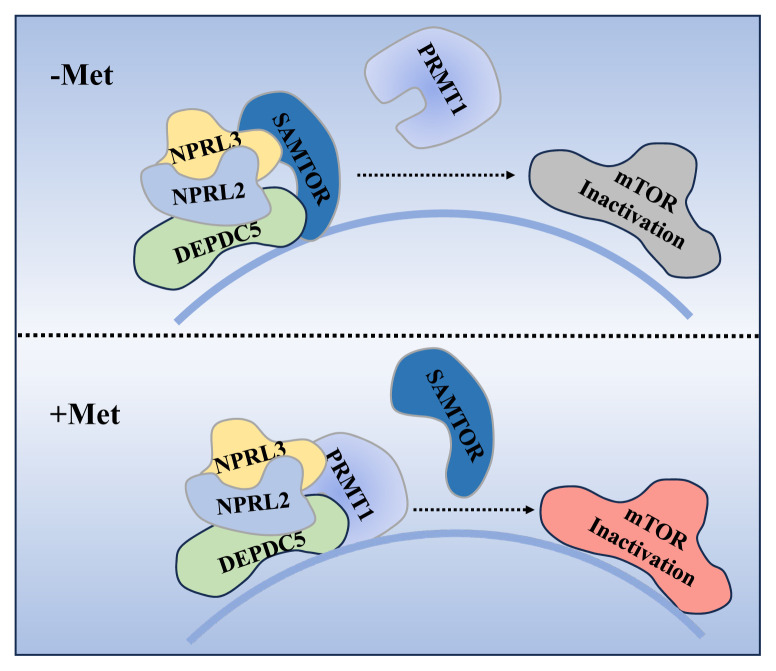
PRMT1 and SAMTOR work together to regulate the methionine-dependent mTORC1 signaling pathway. This figure depicts the process of PRMT1 and SAMTOR jointly regulating and activating mTORC1 signaling pathway under different methionine conditions. In the absence of Met, SAMTOR binds to the complex and inhibits mTOR activation. When Met is sufficient, SAMTOR dissociates from the complex, PRMT1 binds to the complex, and then activates mTOR. mTORC1, mammalian target of rapamycin complex 1; mTOR, mammalian target of rapamycin.

Recent studies have identified SAMTOR, previously known as C7orf60, as an essential component of the mTORC1 signaling pathway, specifically for sensing changes in methionine levels through its ability to bind to S-adenosylmethionine (SAM) [42]. SAMTOR is also known to interact with GATOR1 and KICSTOR, both of which are negative regulators upstream of mTORC1. Consistent with the roles of GATOR1 and KICSTOR, SAMTOR functions similarly as a negative regulator by inhibiting the mTORC1 signaling pathway when overexpressed. In essence, SAMTOR serves as a sensor for SAM, effectively transmitting methionine signals to mTORC1 (Figure 2).

### Threonine sensor

Mitochondrial threonyl-tRNA synthetase (TARS2) activates mTORC 1 in response to changes in threonine levels through an unconventional pathway [43].

TARS2 interacts with deactivated Rags, causing RagA’s GTP load to increase. Threonine supplementation in TARS2-deficient cells is less effective for mTORC1 activation, suggesting that TARS2 is required for mTORC1 activation [44]. This also provides a new direction and theoretical support for the molecular mechanism of amino acids regulation of protein synthesis.

## GENERAL AMINO ACID SENSOR

In addition to the specific amino acid sensor-related pathways mentioned above, many general amino acid sensors have been identified.

### T1R1/T1R3

In addition to the known perceptual mechanisms involving Rag GTPase, which affects mTORC1 activity and lysosome localization, there are also amino acid sensors with mechanisms that are not yet fully understood. These sensors often transmit signals from multiple amino acids. For example, the heterodimer G protein-coupled receptor Tas1r1/Tas1r3 can directly sense amino acid concentrations, and disrupting this receptor alters the localization of mTORC1 [3]. It acts as an amino acid sensor, but notably, it is only active in cells that express taste proteins and/or G proteins specifically involved in taste signaling [45].

### M2PYK

Pyruvate kinase M2 (M2PYK) plays a crucial role in cellular metabolism by rapidly transmitting amino acid signals to stabilize metabolic balance. All amino acids are capable of altering the tetramer conformation of M2PYK by binding to it [46]. This interaction allows M2PYK to be finely regulated by the relative concentrations of amino acids, which is critically important for metabolic rearrangement.

### GCN2

GCN2 is a known amino acid sensor, and ATF4 plays an important role in sensing amino acid deficiency. Recent studies have shown that GCN2 and ATF4 can regulate leucine-deficient WAT Browning by changing neuronal activity [46]. Additionally, when multiple amino acids are depleted, activated GCN2 enhances ATF4 expression. This upregulation leads to the induction of the stress response protein Sestrin2, which is essential for inhibiting mTORC1 by preventing lysosomal activation [47]. These findings indicate that Sestrin2 is a critical mediator in the GCN2 signaling pathway, helping to maintain amino acid homeostasis by suppressing mTORC1 activity. Recent studies have shown that GCN2 is able to promote SARS-CoV-2 receptor ACE2 expression in response to amino acid deprivation. At the same time, amino acid deficiency increases the expression of ACE2, which may exacerbate intestinal SARS-CoV-2 infection [48].

### GPR142

GPR142, a sensor of aromatic amino acids, is predominantly expressed in pancreatic islet cells and intestinal secretory cells. The deletion of GPR142 leads to reduced metabolic activity in mice, indicating its crucial role in mediating hormone secretion in both the pancreas and intestine [49], By sensing amino acids, GPR142 regulates hormone secretion, thereby influencing cellular metabolism.

### DH44

**Figure 3:**
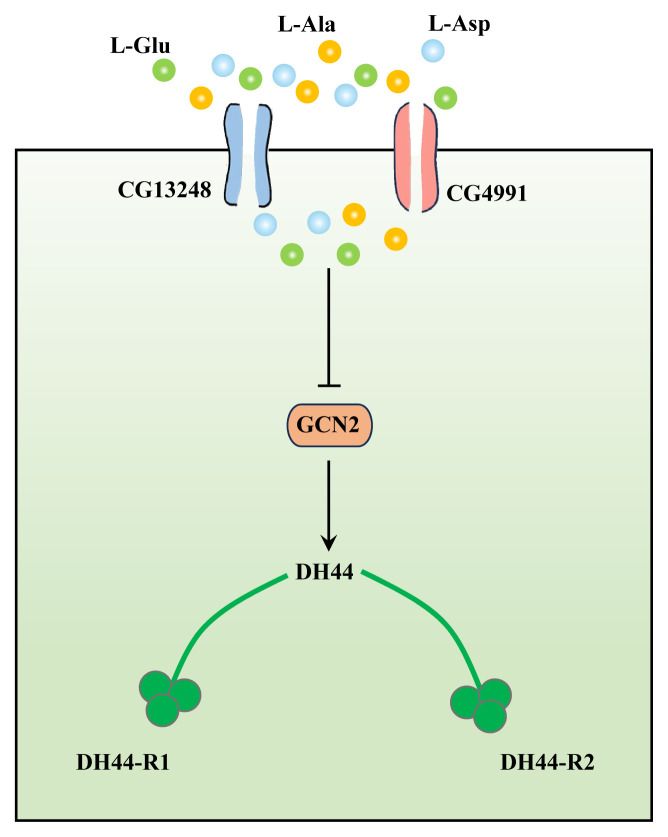
Amino acid stimulation of cell signaling in DH44 neurons. This figure depicts the cell signaling process of DH44 neurons stimulated by L-Glu, L-Ala and L-Asp through the channel proteins CG13248 and CG4991. When the amino acid enters the cell, it inhibits the expression of GCN2, and then transmits the signal to DH44 neurons, and finally to DH44-R1/R2. L-Glu, L-glutamic; L-Ala, L-alanine; L-Asp, L-aspartic.

DH44 is a homolog of mammalian adrenocorticotropin-releasing hormone (CRH), which directly senses amino acids in the diet and promotes food metabolism [50]. The study found that DH44 was able to sense three amino acids in food, L-glutamic acid (L-Glu), L-alanine (L-Ala) and L-aspartic acid (L-Asp), and rapidly promoted food consumption in Drosophila melanogaster (Figure 3). Amino acids in food can quickly and directly activate DH44, suggesting that these neurons are amino acid sensors [51]. Recent studies have suggested that hunger-induced sleep deprivation may be mediated by the brain’s nutrient sensor DH44. But the potential role of nutrition and hunger sensors in regulating sleep and exercise activity is unclear [52].

### Ubr1

The E3 ubiquitin ligase, Ubr1, acts as an amino acid sensor for liver and muscle steatosis and targets Plin2 for amino acid-dependent degradation. In the absence of amino acids, or when the amino acid binding capacity of Ubr1 is reduced, Ubr1-mediated Plin2 degradation is inactivated. As an amino acid sensor regulating protein homeostasis, Ubr1 provides a new direction for the relationship between steatosis and nutritional sensors [53,54].

### ILF3

Interleukin enhancer binding factor 3 (ILF3), encodes a double-stranded RNA (dsRNA) binding protein that combines with other proteins, mRNAs, and dsRNAs to regulate mRNA with stable gene expression [55]. Recent studies have reported identification of ILF3 as a mediator of mTORC1-dependent amino acid induction by CRISPR screening. ILF3 controls mTORC1 by tethering the GATOR complex to the lysosome [56].

## INTERACTION BETWEEN AMINO ACID SENSOR AND GLUCOSE

**Figure 4:**
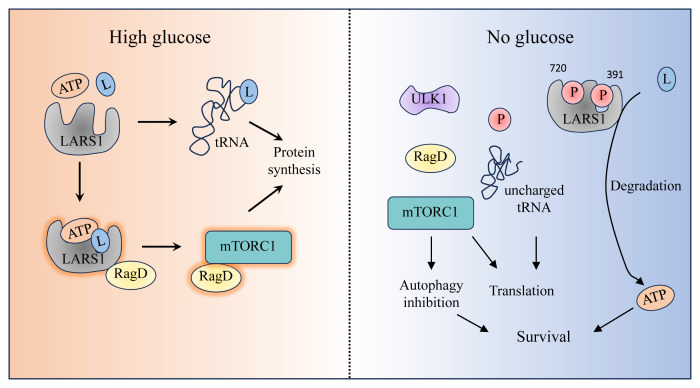
Schematic model of LRS roles in the control of glucose-dependent leucine metabolism. This figure depicts how LRS control the leucine metabolic pathway to promote protein synthesis or cell survival under different glucose conditions. In the condition of sufficient glucose, LARS1 can bind ATP and leucine, and further activate mTOR pathway related proteins, thus promoting protein synthesis. In the absence of glucose, the body’s energy supply is insufficient, and leucine is mainly used as an energy source to maintain cell survival. LRS, leucyl-tRNA synthetase; LARS1, leucyl-tRNA synthetase 1; ATP, adenosine triphosphate; mTOR, mammalian target of rapamycin.

Glucose is a crucial energy source within cells and supports the synthesis of milk proteins, lactose, and fatty acids in mammary gland cells. During lactose synthesis, glucose is transported to the Golgi apparatus by the GLUT1 and GLUT8 transporters, where it contributes to the formation of lactose from UDP-galactose. Additionally, leucine’s utilization within cells is influenced by glucose concentrations; it can be directed towards protein synthesis or diverted to support metabolic energy production [57]. Under conditions of glucose deprivation, ULK1 interferes with the binding capabilities of LRS to leucine, ultimately impeding related protein synthesis. Moreover, even with adequate amino acid supply, a glucose deficit halts translation and reallocates leucine to metabolic processes to generate energy, aiding cell survival [58]. Activated ULK1 phosphorylates LRS at sites 720 and 391, disrupting LARS1-leucine binding and enhancing leucine’s availability for catabolism. Bandyopadhyay et al [59] have shown that cells can adapt to nutrient scarcity by enhancing stress responses, which promotes the recycling or clearance of essential nutrients. In situations where extracellular amino acids are scarce, this adaptation allows cells to store leucine—an essential amino acid—within lysosomes (Figure 4). This storage mechanism enables cells to sequester an essential amino acid needed for protein synthesis, independent of high extracellular amino acid concentrations, with significant quantities of essential amino acids being processed through lysosomes.

In the presence of glucose, LRS can effectively sense leucine, facilitated by glucose, which enhances LRS’s binding to RagD and subsequently activates mTORC1. This activation improves the ability of mammary gland cells to synthesize milk proteins. Specifically, in a high-glucose environment, ATP and leucine bind to LRS, activating it. Once activated, LRS binds to and activates RagD, which in turn binds to and activates mTORC1. The activated mTORC1 then promotes protein synthesis within the cells [60]. Choi et al [61] have discovered that the introduction of BC-LI-0186 inhibits the translocation of RagD and LRS, thereby disrupting their interaction. With the significant reduction in LRS translocation to the lysosome, the activation of mTORC1 by LRS is impaired.

Although the nutrient sensing pathways for glucose and amino acids are crucial, the integration of these nutrient signals remains poorly understood. Recent studies have shown [62] that glucose availability can regulate the central trophic effector mTORC1 via LRS, and glucose deficiency leads to O-GlcNAc acylation at the S1042 residue of LRS. This modification inhibits the binding of LRS to RagD [63]. Within the cell, LRS acts as an initiator switch, hydrolyzing RagD via GTP, which in turn drives the entire Rag GTPase cycle. Meanwhile, the role of Sestrin2 is to ‘shut down’ this cycle by controlling the GTP hydrolysis of RagB on the GTPase-mTORC1 axis [27]. Thus, LRS and Sestrin2 work together to coordinate the GTPase activities of RagD and RagB, ultimately regulating mTORC1 activation.

## INFLUENCE OF AMINO ACID SENSOR ON LIVESTOCK PRODUCTION

Amino acids are an important part of livestock and poultry diets, and amino acid sensors can participate in the growth and production activities of livestock and poultry. It has been reported that the improvement in growth performance caused by the addition of individual essential amino acids to rats on a low protein diet is not solely attributable to the increased availability of essential amino acids to the body [64]. In addition, different essential amino acids supplementation in low crude protein diet has a great effect on the growth performance of weaned piglets [65]. It has also been reported that dietary supplementation with leucine or its metabolites can stimulate protein synthesis in skeletal muscle of piglets. Leucine is a mechanistic target by stimulating mTORC1 phosphorylation in pig muscle. This activation process is achieved through the dissociation of the leucine sensor Sestrin2 from the GATOR2 complex and the binding of Rag A/C to mTOR [66]. Recently, Leucine sensor SESN2 was found to be a protective regulator of endoplasmic reticulum (ER) stress in porcine oocytes during *in vitro* maturation on the one hand, defects in SESN2 can block the activity of nuclear erythrocyte 2-associated factor 2 (NRF2) on the spindle assembly of porcine oocytes. At the same time, the SESN20-NRF2 signaling pathway may play an important role in ER induction during meiosis and oocyte maturation [67].

Other studies have shown that *T1R1* knockdown can reduce the expression of β-casein in HC11 cells in complete medium. T1R1 can regulate the expression of lactation-related proteins through mTOR signaling pathway, and thus participate in milk protein synthesis [68]. Interference with *T1R1* can alter the localization of mTORC1, up-regulate key amino acid transporters, and induce autophagy. These studies reveal the mechanism by which amino acid availability is communicated to mTORC1 in mammals via the G protein-coupled receptor T1R1/T1R3. This may provide a new way for amino acid receptors to further participate in milk protein synthesis [3]. Under heat stress, arginine activates the mTORC1 pathway through its receptor CASTOR1, thereby alleviating mammary gland damage, suggesting that ingestion of arginine feed or feed additives is a way to improve milk production in dairy cows at high temperatures [69].

It has been reported that the changes of liver GCN2/eIF2α/ATF4 pathway were studied by restricting the intake of nitrogen and phosphorus in the diet of young goats, and increased mRNA and protein expression of liver GCN2 was observed [70]. In addition, recent studies have found that acetyl-CoA levels in the native Chinese pig breed Yacha (YC) are significantly lower than those in the Western pig breed Yorkshire (YS), suggesting that acetyl-CoA may be one explanation for the differences in growth and development between YC and YS breeds [71]. The scientists found that when fed a high-grain (HG) diet, the expression of sensory receptors GPR43 and T1R1 was increased in the ileum of goats. This explains the adaptation of the goat’s GI tract to the HG diet and promotes its absorption of glucose, fatty acids and amino acids [72]. The molecular mechanism of amino acid sensor involved in livestock production has not yet been elucidated, and whether it is to improve the quality of livestock meat or milk production, it is crucial for economic development.

## PERSPECTIVE

The perception of nutrients is crucial for cell growth, with amino acids playing a vital role in mammalian metabolism. While the mechanism by which mTORC1 senses leucine is well-studied, other amino acid sensing pathways require further exploration. Beyond the well-understood molecular binding mechanisms of arginine sensors like CASTOR1/2 and the leucine sensor Sestrin2, the structures of other sensors or their complexes with binding proteins need more detailed analysis to clarify their functions. It remains to be confirmed whether there are additional amino acid sensors beyond those discussed here, and their specific sensing mechanisms are yet to be fully elucidated. Moreover, although numerous amino acid receptors have been identified as part of the mTORC1 signaling pathway, precisely how mTORC1 integrates these signals is still an unresolved question. Currently known amino acids, including leucine, arginine and so on, can participate in the regulation of cell growth and protein synthesis through the mTOR signaling pathway. In addition, most amino acids can enter the cell via SLC7A5/SLC3A2, and then activate the mTOR pathway via SLC38A9-Ragulator pathway. At present, the methods commonly used to determine free amino acids *in vivo* are mainly detected by enzyme-linked immunosorbent assay kit or high-throughput sequencing, and amino acids can also be labeled by stable isotope method.

Furthermore, studying the molecular mechanisms underlying the differences in growth and development among varieties can help to achieve higher disease resistance, higher lean meat percentage and faster growth rate. Exploring amino acid sensor can help to explore amino acid transport in domestic animals, and thus promote the rate of amino acid absorption in domestic animals, which is of great significance for the health and production of domestic animals. Although some amino acid sensors have been studied well enough, the majority of amino acid sensors are still unproven, and new amino acid sensors, whether from cells or animal tissues, need to be discovered. In addition, mTOR’s nutrition-sensing mechanism plays a role in food metabolism and food selection, and further study of mTOR’s nutrition-sensing mechanism has great significance for improving animal husbandry growth and reproduction. Going forward, elucidating the specific regulatory mechanisms of amino acids holds significant potential to enhance both the yield and quality of livestock products.

## References

[1] Layman DK, Anthony TG, Rasmussen BB (2015). Defining meal requirements for protein to optimize metabolic roles of amino acids. Am J Clin Nutr.

[2] Yan L, Mieulet V, Burgess D (2010). PP2A T61 epsilon is an inhibitor of MAP4K3 in nutrient signaling to mTOR. Mol Cell.

[3] Wauson EM, Zaganjor E, Lee AY (2012). The G protein-coupled taste receptor T1R1/T1R3 regulates mTORC1 and autophagy. Mol Cell.

[4] Caron E, Ghosh S, Matsuoka Y (2010). A comprehensive map of the mTOR signaling network. Mol Syst Biol.

[5] Yoon MS (2017). The role of mammalian target of rapamycin (mTOR) in insulin signaling. Nutrients.

[6] Rhoads JM, Niu X, Odle J, Graves LM (2006). Role of mTOR signaling in intestinal cell migration. Am J Physiol Gastrointest Liver Physiol.

[7] Yao K, Yin YL, Chu W (2008). Dietary arginine supplementation increases mTOR signaling activity in skeletal muscle of neonatal pigs. J Nutr.

[8] Xia J, Wang R, Zhang T, Ding J (2016). Structural insight into the arginine-binding specificity of CASTOR1 in amino acid-dependent mTORC1 signaling. Cell Discov.

[9] Saxton RA, Chantranupong L, Knockenhauer KE, Schwartz TU, Sabatini DM (2016). Mechanism of arginine sensing by CASTOR1 upstream of mTORC1. Nature.

[10] Rebsamen M, Superti-Furga G (2016). SLC38A9: A lysosomal amino acid transporter at the core of the amino acid-sensing machinery that controls MTORC1. Autophagy.

[11] Wyant GA, Abu-Remaileh M, Wolfson RL (2017). mTORC1 Activator SLC38A9 is required to efflux essential amino acids from lysosomes and use protein as a nutrient. Cell.

[12] Jung J, Genau HM, Behrends C (2015). Amino acid-dependent mTORC1 regulation by the lysosomal membrane protein SLC38A9. Mol Cell Biol.

[13] Lee IP, Evans AK, Yang C (2014). Toxoplasma gondii is dependent on glutamine and alters migratory profile of infected host bone marrow derived immune cells through SNAT2 and CXCR4 pathways. PLoS One.

[14] Hyde R, Cwiklinski EL, MacAulay K, Taylor PM, Hundal HS (2007). Distinct sensor pathways in the hierarchical control of SNAT2, a putative amino acid transceptor, by amino acid availability. J Biol Chem.

[15] Pinilla J, Aledo JC, Cwiklinski E, Hyde R, Taylor PM, Hundal HS (2011). SNAT2 transceptor signalling via mTOR: a role in cell growth and proliferation?. Front Biosci (Elite Ed).

[16] Shen K, Sabatini DM (2018). Ragulator and SLC38A9 activate the Rag GTPases through noncanonical GEF mechanisms. Proc Natl Acad Sci USA.

[17] Huttlin EL, Ting L, Bruckner RJ (2015). The bioplex network: A systematic exploration of the human interactome. Cell.

[18] Bar-Peled L, Chantranupong L, Cherniack AD (2013). A Tumor suppressor complex with GAP activity for the Rag GTPases that signal amino acid sufficiency to mTORC1. Science.

[19] Chantranupong L, Scaria SM, Saxton RA (2016). The CASTOR proteins are arginine sensors for the mTORC1 pathway. Cell.

[20] Tang Y, Tan B, Li G, Li J, Ji P, Yin Y (2018). The regulatory role of MeAIB in protein metabolism and the mtor signaling pathway in porcine enterocytes. Int J Mol Sci.

[21] Jung JW, Macalino S, Cui M (2019). Transmembrane 4 l six family member 5 senses arginine for mTORC1 signaling. Cell Metab.

[22] He XD, Gong W, Zhang JN (2018). Sensing and transmitting intracellular amino acid signals through reversible lysine aminoacylations. Cell Metab.

[23] Chantranupong L, Wolfson RL, Orozco JM (2014). The Sestrins interact with GATOR2 to negatively regulate the amino-acid-sensing pathway upstream of mTORC1. Cell Rep.

[24] Li XZ, Yan XH (2019). Sensors for the mTORC1 pathway regulated by amino acids. J Zhejiang Univ SciB.

[25] Parmigiani A, Nourbakhsh A, Ding B (2014). Sestrins inhibit mTORC1 kinase activation through the GATOR complex. Cell Rep.

[26] Peng M, Yin N, Li MO (2014). Sestrins function as guanine nucleotide dissociation inhibitors for Rag GTPases to control mTORC1 signaling. Cell.

[27] Lee M, Kim JH, Yoon I (2018). Coordination of the leucine-sensing Rag GTPase cycle by leucyl-tRNA synthetase in the mTORC1 signaling pathway. Proc Natl Acad Sci USA.

[28] Kim YM, Stone M, Hwang TH (2012). SH3BP4 is a negative regulator of amino acid-Rag GTPase-mTORC1 signaling. Mol Cell.

[29] Luo C, Zheng N, Zhao S, Wang J (2019). Sestrin2 negatively regulates casein synthesis through the SH3BP4-mTORC1 pathway in response to aa depletion or supplementation in cow mammary epithelial cells. J Agric Food Chem.

[30] Tome D (2004). Protein, amino acids and the control of food intake. Br J Nutr.

[31] Budanov AV, Karin M (2008). P53 target genes sestrin1 and sestrin2 connect genotoxic stress and mTOR signaling. Cell.

[32] Guttler A, Weinholdt C, Ruff E (2023). SESN2 knockdown increases betulinic acid-induced radiosensitivity of hypoxic breast cancer cells. Cells.

[33] Ma Y, Zhang G, Kuang Z (2022). Empagliflozin activates Sestrin2-mediated AMPK/mTOR pathway and ameliorates lipid accumulation in obesity-related nonalcoholic fatty liver disease. Front Pharmacol.

[34] Cusack S, Yaremchuk A, Tukalo M (2000). The 2 A crystal structure of leucyl-tRNA synthetase and its complex with a leucyl-adenylate analogue. EMBO J.

[35] Han JM, Jeong SJ, Park MC (2012). Leucyl-tRNA synthetase is an intracellular leucine sensor for the mTORC1-signaling pathway. Cell.

[36] Chen X, Ma JJ, Tan M (2011). Modular pathways for editing non-cognate amino acids by human cytoplasmic leucyl-tRNA synthetase. Nucleic Acids Res.

[37] Jewell JL, Russell RC, Guan KL (2013). Amino acid signalling upstream of mTOR. Nat Rev Mol Cell Biol.

[38] Kimball SR, Gordon BS, Moyer JE, Dennis MD, Jefferson LS (2016). Leucine induced dephosphorylation of Sestrin2 promotes mTORC1 activation. Cell Signal.

[39] Son SM, Park SJ, Lee H (2019). Leucine signals to mTORC1 via its metabolite acetyl-coenzyme A. Cell Metab.

[40] Zhang YK, Qu YY, Lin Y (2017). Enoyl-CoA hydratase-1 regulates mTOR signaling and apoptosis by sensing nutrients. Nat Commun.

[41] Kim S, Snyder SH (2011). Nutrient amino acids signal to mTOR via inositol polyphosphate multikinase. Cell Cycle.

[42] Gu X, Orozco JM, Saxton RA (2017). SAMTOR is an S-adenosylmethionine sensor for the mTORC1 pathway. Science.

[43] Kim SH, Choi JH, Wang P (2021). Mitochondrial threonyl-tRNA synthetase TARS2 is required for threonine-sensitive mTORC1 activation. Mol Cell.

[44] Zeng QY, Zhang F, Zhang JH (2023). Loss of threonyl-tRNA synthetase-like protein Tarsl2 has little impact on protein synthesis but affects mouse development. J Biol Chem.

[45] Muroi Y, Ishii T (2012). Umami taste receptor functions as an amino acid sensor via Gαs subunit in N1E-115 neuroblastoma cells. J Cell Biochem.

[46] Yuan M, McNae IW, Chen Y (2018). An allostatic mechanism for M2 pyruvate kinase as an amino-acid sensor. Biochem J.

[47] Ye J, Palm W, Peng M (2015). GCN2 sustains mTORC1 suppression upon amino acid deprivation by inducing Sestrin2. Genes Dev.

[48] Hu X, Niu Y, Luo P (2022). Amino acid sensor GCN2 promotes SARS-CoV-2 receptor ACE2 expression in response to amino acid deprivation. Commun Biol.

[49] Rudenko O, Shang J, Munk A (2019). The aromatic amino acid sensor GPR142 controls metabolism through balanced regulation of pancreatic and gut hormones. Mol Metab.

[50] Chen YD, Dahanukar A (2018). DH44 neurons: gut-brain amino acid sensors. Cell Res.

[51] Yang Z, Huang R, Fu X (2018). A post-ingestive amino acid sensor promotes food consumption in Drosophila. Cell Res.

[52] Oh Y, Suh G (2023). Starvation-induced sleep suppression requires the Drosophila brain nutrient sensor. J Neurogenet.

[53] Zhao W, Zhang Y, Lin S (2023). Identification of Ubr1 as an amino acid sensor of steatosis in liver and muscle. J Cachexia Sarcopenia Muscle.

[54] Xia Z, Webster A, Du F, Piatkov K, Ghislain M, Varshavsky A (2008). Substrate-binding sites of UBR1, the ubiquitin ligase of the N-end rule pathway. J Biol Chem.

[55] Li K, Wu JL, Qin B (2020). ILF3 is a substrate of SPOP for regulating serine biosynthesis in colorectal cancer. Cell Res.

[56] Yan G, Yang J, Li W, Guo A, Guan J, Liu Y (2023). Genome-wide CRISPR screens identify ILF3 as a mediator of mTORC1-dependent amino acid sensing. Nat Cell Biol.

[57] Kim BG (2022). Insects as animal feed: novel ingredients for use in pet, aquaculture and livestock diet. Anim Biosci.

[58] Yoon I, Nam M, Kim HK (2019). Glucose-dependent control of leucine metabolism by leucyl-tRNA synthetase 1. Science.

[59] Bandyopadhyay U, Todorova P, Pavlova NN (2022). Leucine retention in lysosomes is regulated by starvation. Proc Natl Acad Sci USA.

[60] Kim S, Yoon I, Son J (2021). Leucine-sensing mechanism of leucyl-tRNA synthetase 1 for mTORC1 activation. Cell Rep.

[61] Choi H, Son JB, Kang J (2017). Leucine-induced localization of Leucyl-tRNA synthetase in lysosome membrane. Biochem Biophys Res Commun.

[62] Kim JH, Lee C, Lee M (2017). Control of leucine-dependent mTORC1 pathway through chemical intervention of leucyl-tRNA synthetase and RagD interaction. Nat Commun.

[63] Kim K, Yoo HC, Kim BG (2022). O-GlcNAc modification of leucyl-tRNA synthetase 1 integrates leucine and glucose availability to regulate mTORC1 and the metabolic fate of leucine. Nat Commun.

[64] Liu W, Wang T, Zhao K (2024). Effects of individual essential amino acids on growth rates of young rats fed a low-protein diet. Animals.

[65] Wellington MO, Hulshof TG, Resink JW, Ernst K, Balemans A, Page GI (2023). The effect of supplementation of essential amino acid combinations in a low crude protein diet on growth performance in weanling pigs. Transl Anim Sci.

[66] Suryawan A, Rudar M, Fiorotto ML, Davis TA (2020). Differential regulation of mTORC1 activation by leucine and beta-hydroxy-beta-methylbutyrate in skeletal muscle of neonatal pigs. J Appl Physiol.

[67] Park HJ, Yang SG, Koo DB (2022). SESN2/NRF2 signaling activates as a direct downstream regulator of the PERK pathway against endoplasmic reticulum stress to improve the in vitro maturation of porcine oocytes. Free Radic Biol Med.

[68] Wang Y, Liu J, Wu H, Fang X, Chen H, Zhang C (2017). Amino acids regulate mTOR pathway and milk protein synthesis in a mouse mammary epithelial cell line is partly mediated by T1R1/T1R3. Eur J Nutr.

[69] Gai Z, Hu S, He Y (2024). L-arginine alleviates heat stress-induced mammary gland injury through modulating CASTOR1-mTORC1 axis mediated mitochondrial homeostasis. Sci Total Environ.

[70] Weber SL, Hustedt K, Schnepel N, Visscher C, Muscher-Banse AS (2023). Modulation of GCN2/eIF2alpha/ATF4 pathway in the liver and induction of FGF21 in young goats fed a protein- and/or phosphorus-reduced diet. Int J Mol Sci.

[71] Tao X, Kong FJ, Liang Y (2023). Screening of candidate genes related to differences in growth and development between Chinese indigenous and Western pig breeds. Physiol Genomics.

[72] Tian C, Wu J, Jiao J, Zhou C, Tan Z (2022). The expression of nutrient chemosensing gate molecules in the ileum and colon is altered for goats fed on a high-grain diet. Anim Sci J.

